# Genome-wide association studies of plant architecture-related traits and 100-seed weight in soybean landraces

**DOI:** 10.1186/s12863-021-00964-5

**Published:** 2021-03-06

**Authors:** Xiaoli Zhang, Wentao Ding, Dong Xue, Xiangnan Li, Yang Zhou, Jiacheng Shen, Jianying Feng, Na Guo, Lijuan Qiu, Han Xing, Jinming Zhao

**Affiliations:** 1 National Center for Soybean Improvement, Key Laboratory of Biology and Genetics and Breeding for Soybean, Ministry of Agriculture, State Key Laboratory for Crop Genetics and Germplasm Enhancement, College of Agriculture, Nanjing Agricultural University, Nanjing, 210095 China; 2 The National Key Facility for Crop Gene Resources and Genetic Improvement (NFCRI), Key Lab of Germplasm Utilization (MOA), Institute of Crop Science, Chinese Academy of Agricultural Sciences, Beijing, 100081 China

**Keywords:** Soybean (*Glycine max* (L.) Merr.), Plant architecture-related traits, 100-seed weight, GWAS, Candidate genes

## Abstract

**Background:**

Plant architecture-related traits (e.g., plant height (PH), number of nodes on main stem (NN), branch number (BN) and stem diameter (DI)) and 100-seed weight (100-SW) are important agronomic traits and are closely related to soybean yield. However, the genetic basis and breeding potential of these important agronomic traits remain largely ambiguous in soybean (*Glycine max* (L.) Merr.).

**Results:**

In this study, we collected 133 soybean landraces from China, phenotyped them in two years at two locations for the above five traits and conducted a genome-wide association study (GWAS) using 82,187 single nucleotide polymorphisms (SNPs). As a result, we found that a total of 59 SNPs were repeatedly detected in at least two environments. There were 12, 12, 4, 4 and 27 SNPs associated with PH, NN, BN, DI and 100-SW, respectively. Among these markers, seven SNPs (AX-90380587, AX-90406013, AX-90387160, AX-90317160, AX-90449770, AX-90460927 and AX-90520043) were large-effect markers for PH, NN, BN, DI and 100-SW, and 15 potential candidate genes were predicted to be in linkage disequilibrium (LD) decay distance or LD block. In addition, real-time quantitative PCR (qRT-PCR) analysis was performed on four 100-SW potential candidate genes, three of them showed significantly different expression levels between the extreme materials at the seed development stage. Therefore, *Glyma.05 g127900*, *Glyma.05 g128000* and *Glyma.05 g129000* were considered as candidate genes with 100-SW in soybean.

**Conclusions:**

These findings shed light on the genetic basis of plant architecture-related traits and 100-SW in soybean, and candidate genes could be used for further positional cloning.

**Supplementary Information:**

The online version contains supplementary material available at 10.1186/s12863-021-00964-5.

## Background

Soybean [*Glycine max* (L.) Merr.] is an important economic and oil crop, providing abundant plant proteins and oil to humans [1]. Researchers have increased soybean yield as much as possible through traditional breeding and molecular breeding methods [2]. The effort to meet soybean demand on existing cropland areas for a global population of 9.7 billion by the year 2050 puts pressure on narrowing the existing gap between the average yield and yield potential [3, 4]. Plant breeders continually research how to maximize soybean yield to solve the contradiction between supply and demand [5]. Plant architecture is a key factor affecting planting density and grain yield in soybean. The ideal soybean plant architecture optimizes the canopy architecture, improves photosynthetic efficiency, and prevents lodging, thus resulting in high overall grain yield [5, 6]. 100-seed weight (100-SW) is an important component of soybean yield and an important target trait in field breeding [7]. Moreover, larger seeds, which have greater energy stores, may improve seedling establishment [8]. Given the importance of four plant architecture-related traits (plant height (PH), number of nodes on main stem (NN), branch number (BN) and stem diameter (DI)) and 100-SW of soybean, a large number of QTLs associated with these traits have been identified in the past decade [9], but the genes underlying the QTLs and their functions remain largely unknown.

Plant architecture-related traits and 100-SW of soybean are complex quantitative traits influenced by multiple QTLs and are susceptible to environmental factors [5]. Previous studies were conducted to dissect the genetic basis of plant architecture-related traits and 100-SW in biparental populations. Hundreds of QTLs were detected across the whole genome of soybean, with many being simultaneously detected in multiple populations [10–13]. These studies demonstrated that the genetic mapping of quantitative traits using genetic linkage maps is an efficient approach for identifying QTLs. Currently, numerous researchers use molecular markers to identify QTLs controlling these important agronomic traits [14]. Given the increased use of molecular markers to identify QTLs, opportunities exist to significantly increase our knowledge of the genetic basis of these traits and to accelerate soybean breeding [15]. To date, many QTLs for plant architecture-related traits and 100-SW have been reported in investigations using biparental populations [11, 16–18]. According to the SoyBase database (http://www.soybase.org), there are 239 QTLs controlling PH in soybean, which are distributed on 20 chromosomes, and 37 QTLs related to NN. For BN and 100-SW, 21 and 297 related QTLs have been reported, respectively. And there were a few reports on the QTL position of DI in soybean. Despite the extensive QTL analysis on plant architecture-related traits and 100-SW of soybean, traditional biparent segregation populations have several disadvantages, including limited genetic variation and mapping resolution [19].

With the development of genotyping and sequencing technologies, the pace of genetic research on crop quantitative traits has been accelerated. Comparing with bi-parental QTL mapping studies, the genome-wide association study (GWAS) is a more powerful method for dissecting the QTLs underlying agronomically important traits in natural populations. High density of markers in the GWAS also enables one to predict or identify causal genes [20]. In recent years, GWAS has rapidly became a popular and powerful tool to detect natural variation that accounts for complex and important agronomic traits of crops, and has been successfully applied to the studies of many crops, such as *Arabidopsis thaliana* [21], rice [22, 23], maize [24, 25], soybean [9], and foxtail millet [26]. In soybean, the evaluation of several specific agronomic traits, including seed protein content and oil concentration [27, 28], sudden death syndrome resistance [29], cyst nematode resistance [30, 31], and flowering time [32], were conducted through GWAS by genotyping either with Illumina Bead Chips or specific locus amplified fragment sequence. These studies provide valuable resources for the future molecular breeding of soybean.

In recent years, association studies have been performed in grain soybean for plant architecture and yield-related traits, and they have achieved great success in identifying loci with high mapping precision [33]. Through genomic consequences of selection and GWAS, a total of 125 candidate selection regions were identified of 9 agronomic traits and 5 potential candidate genes were predicted [34]. Zhang et al. (2016) conducted a genome-wide association study in a population of 309 soybean germplasm accessions, identified 22 loci of minor effect and predicted 3 candidate genes on chromosome 19 [35]. Fang et al. (2017) collected 809 soybean materials worldwide and performed a two-year phenotypic determination of 84 agronomic traits in three locations, and identified 245 SNPs, including known genes such as *Dt1*, *E2*, *E1*, *Ln*, *Dt2*, *Fan* and *Fap*, as well as 16 unreported loci, which are pleiotropic for different traits [9]. Diers et al. (2018) performed an association mapping for the NAM population of 5600 inbred lines, and SNP data revealed 23 significant marker-trait associations for yield, 19 for maturity, 15 for plant height, 17 for plant lodging, and 29 for seed mass [36]. Association mapping has been used to identify significantly associated locus for flowering stage, grain filling stage, maturity stage, yield and 100-SW of soybean, and detected nine, six, four, five and two significantly associated SNPs, respectively [37]. A total of 58 SNPs that were significantly associated with internode number (IN), plant height (PH), seed weight (SW), and seed yield per plant (SYP) were identified by GWAS, and 28 related candidate genes were predicted [38]. By using GWAS, 14 quantitative trait nucleotides (QTNs) were identified to be associated with seed length, 13 with seed width and 21 with seed thickness in four tested environments [39]. Using the multilocus GWAS methods, a total of 118 QTNs of 100-seed weight were detected, and three potential candidate genes were identified in soybean [40]. Although a lot of researches for plant architecture and yield-related traits have been carried out in soybean, the molecular mechanism underlying these traits in soybean remains unclear due to their complexity genetic mechanism.

In this study, we collected 133 diverse soybean landraces, cultivated them at two locations for 2 years, and phenotyped them for the four plant architecture-related traits (PH, NN, BN and DI) and 100-SW. Using the 180 K AXIOM SoyaSNP array, more than 160 thousand genetic markers were generated. After filtering and quality control, a total of 82,187 high-quality SNPs (MAF > 0.05, missing data < 10%) were used for association mapping. The endeavor from comprehensive GWAS analyses enabled the identification of the underlying genetic loci and prediction of potential candidate genes for five traits. In addition, candidate genes of 100-SW were initially confirmed by qRT-PCR. The objectives of this study were to reveal the genetic basis of plant architecture-related traits and 100-SW in soybean and provide valuable markers and candidate genes for the molecular breeding of soybean.

## Results

### Phenotypic analysis of the four plant architecture-related traits and 100-SW

**Fig. 1:**
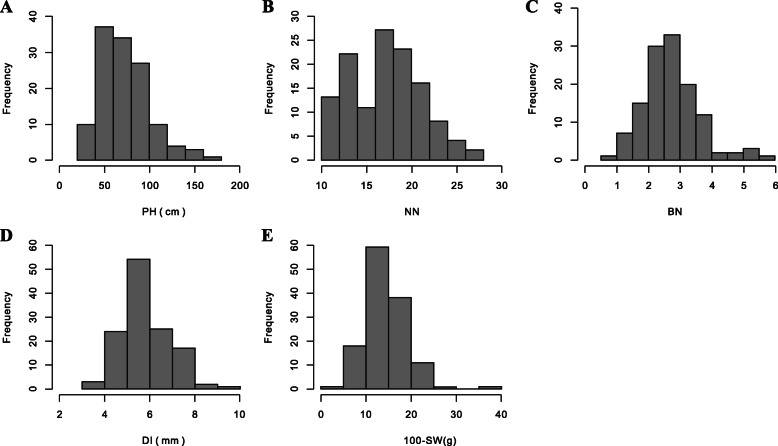
Phenotypic variations of the four plant architecture-related traits and 100-SW in soybean landraces. **a**, **b**, **c**, **d** and **e** represent the frequency distribution of PH, NN, BN, DI and 100-SW, respectively

Four plant architecture-related traits and 100-SW were investigated using the 133 soybean landraces planted in two consecutive years at two locations. Extensive phenotypic variations were observed for all traits in the 133 soybean landraces (Table 1). The phenotypic variation of PH, NN, BN and DI in the 2016JP, 2017JP and 2017DT environments were 21.64–249.33 cm, 9.11–28.83, 0–7.33 and 2.90–11.01 mm, respectively. The 100-SW ranged from 3.76 to 37.23 g in the 2017JP and 2017DT environments. The average of PH in 2017DT was higher than that in 2016JP and 2017JP, whereas all of the other traits revealed little variation (Table 1). The frequency distribution of the five traits based on best linear unbiased prediction (BLUP) values displayed an approximately normal distribution, except for a few materials that had large deviations (Fig. 1). Analysis of variance indicated that the genotype (G), environment (E) and genotype by environment interaction (G × E) had significant effects on PH, NN and DI (*P* < 0.01; Table 1). The genotype (G) and genotype by environment interaction (G × E) had significant effects on BN and 100-SW, but the genotype by environment interaction (G × E) had no significant effects. Heritability (*h*^*2*^) was calculated for the four plant architecture-related traits and 100-SW (Table 1). The heritabilities of the five traits ranged from 65.17 to 98.66%. Among them, the heritability of 100-SW was the highest at 98.66%, while the heritability of BN was the lowest at 65.17%. The correlation coefficients for the five traits were calculated based on the BLUP values and are summarized in Table 2. There was a significant positive correlation between PH and NN, with a correlation coefficient of 0.894. There was also a significant positive correlation between PH, NN, BN and DI. Additionally, 100-SW was only significantly positively correlated with DI, with a correlation coefficient of 0.244. Correlation analysis showed that there was a positive correlation between PH, NN, BN, DI and 100-SW in soybean.

**Table 1:** Descriptive statistics, ANOVA and heritability (*h*^2^) for the four plant architecture-related traits and 100-SW across multiple environments

Traits^a^	Environments^b^	Mean	*SD*^c^	Min	Max	Skew	Kurtosis	G^d^	E^d^	G × E^d^	*h*^2 e^(%)
PH	2016JP	45.43	15.73	21.64	95.56	0.96	0.23	**	**	**	88.85
	2017JP	74.67	30.51	27.11	184.33	0.91	0.76				
	2017DT	100.78	38.37	24.00	249.33	0.72	1.15				
NN	2016JP	15.54	3.96	9.11	28.00	0.75	0.03	**	**	**	93.53
	2017JP	17.87	4.39	10.00	28.33	0.16	−0.90				
	2017DT	18.41	4.17	10.00	28.83	−0.05	−0.64				
BN	2016JP	2.83	1.16	0.56	7.33	0.85	1.70	**		**	65.17
	2017JP	2.68	1.14	0.00	6.00	0.20	0.15				
	2017DT	2.72	1.25	0.17	6.67	0.83	1.38				
DI	2016JP	4.57	0.94	2.90	8.85	1.09	2.64	**	**	**	67.39
	2017JP	6.20	1.29	3.86	9.56	0.66	−0.09				
	2017DI	6.83	1.27	3.74	11.01	0.28	0.34				
100-SW	2017JP	14.44	4.29	5.34	34.25	0.97	2.59	**		**	98.66
	2017DI	14.43	4.55	3.76	37.23	1.09	4.00				

^a^PH (Plant height), NN (Number of nodes on main stem), BN (Branch number), DI (Stem diameter) and 100-SW (100-seed weight)

^b^2016JP, 2017JP and 2017DT represent the environments of Jiangpu in 2016, Jiangpu in 2017 and Dangtu in 2017, respectively

^c^*SD* represents standard deviation

^d^G, E and G × E represent the effect for genotype, environment and genotype × environment interaction, respectively. **Significant at *P* ≤ 0.01

^e^*h*^2^ (%) represents heritability

**Table 2:** Correlation coefficients among the four plant architecture-related traits and 100-SW

	PH	NN	BN	DI
NN	0.894^**^			
BN	0.400^**^	0.490**		
DI	0.482^**^	0.544**	0.460**	
100-SW	0.044	−0.031	0.013	0.244**

The values represent phenotypic correlation coefficients based on the BLUP values across multiple environments. *PH* Plant height, *NN* Number of nodes on main stem, *BN* Branch number, *DI* Stem diameter and *100-SW* 100-seed weight. ** Significant at *P* ≤ 0.01

### Genetic diversity*,* LD and population structure

Analyses of the SNP data, LD and population structure used in this study were reported by genotyping the 133 soybean landraces with the 180 K AXIOM Soya SNP array [41]. According to MAF > 0.05 and missing data < 10%, we detected a total of 82,187 SNPs for subsequent analysis. The marker density ranged from 16.28 kb/SNP to 9.57 kb/SNP, with an average of 11.76 kb/SNP. The average LD decay of all chromosomes was 119.07 kb at the *r*^*2*^ calculated via PLINK V1.07 (Additional file 1: Fig. S1) [41]. Previous studies have used 8270 SNPs and STRUCTURE 2.3.4 software to analyze population structure of the population of the 133 soybean landraces [41]. Population structure analysis showed that the mean LnP (K) did not plateau at a single K value, but instead continued to increase with relatively constant increments. Calculation of Delta K revealed a sharp peak at K = 2, therefore, the 133 soybean landraces were divided into two subgroups, designated subgroup 1 and subgroup 2 (Additional file 2: Fig. S2) [41].

### Model comparison for controlling false associations

**Fig. 2:**
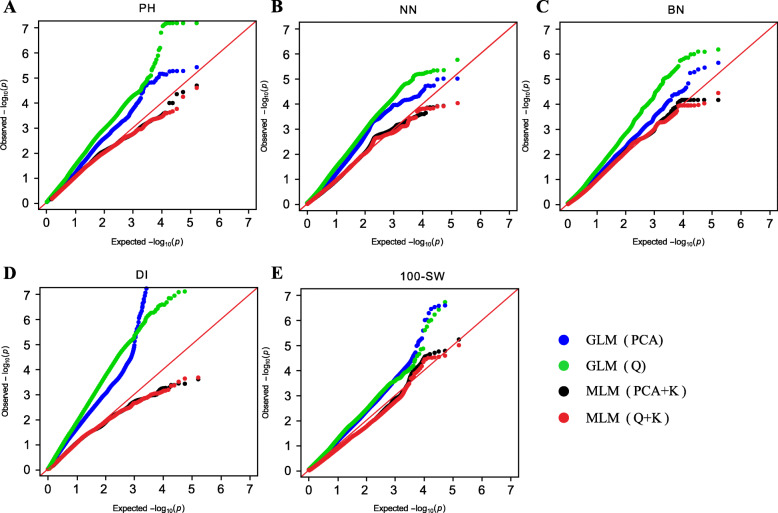
Q-Q plots of the estimated -log_10_(*P*) from association mapping of the four plant architecture-related traits and 100-SW. **a**, **b**, **c**, **d**, and **e** represent Q-Q plots for PH, NN, BN, DI and 100-SW based on the BLUP values across multiple environments, respectively. The red line bisecting the plot represents the expected *P* values with no associations present. The blue line represents observed *P* values using the GLM (PCA) model. The green line represents observed *P* values using the GLM (Q) model. The black line represents observed *P* values using the MLM (PCA + K) model. The red line represents observed *P* values using the MLM (Q + K) model

Association mapping for the four plant architecture-related traits and 100-SW were performed to evaluate the effects of population structure (Q), principal component analysis (PCA) and familial relationship (*K*) on controlling false associations. For the five traits, the observed *P* values from the GLM (PCA) and GLM (Q) models greatly deviated from the expected *P* values assuming that no association existed. The *P* values from the MLM (PCA + K) and MLM (Q + K) models were similar and close to the expected *P* values (Fig. 2). Although the MLM (PCA + K) model detected fewer associations than the MLM (Q + K) model, the observed *P* values for the Q + K model were closer to the expected *P* values than the MLM (PCA + K) model, indicating that the MLM (Q + K) model could effectively control false positive associations and avoid false negative associations. Therefore, in the current study, the MLM (Q + K) model was chosen for association mapping.

### Association mapping of the four plant architecture-related traits and 100-SW

**Fig. 3:**
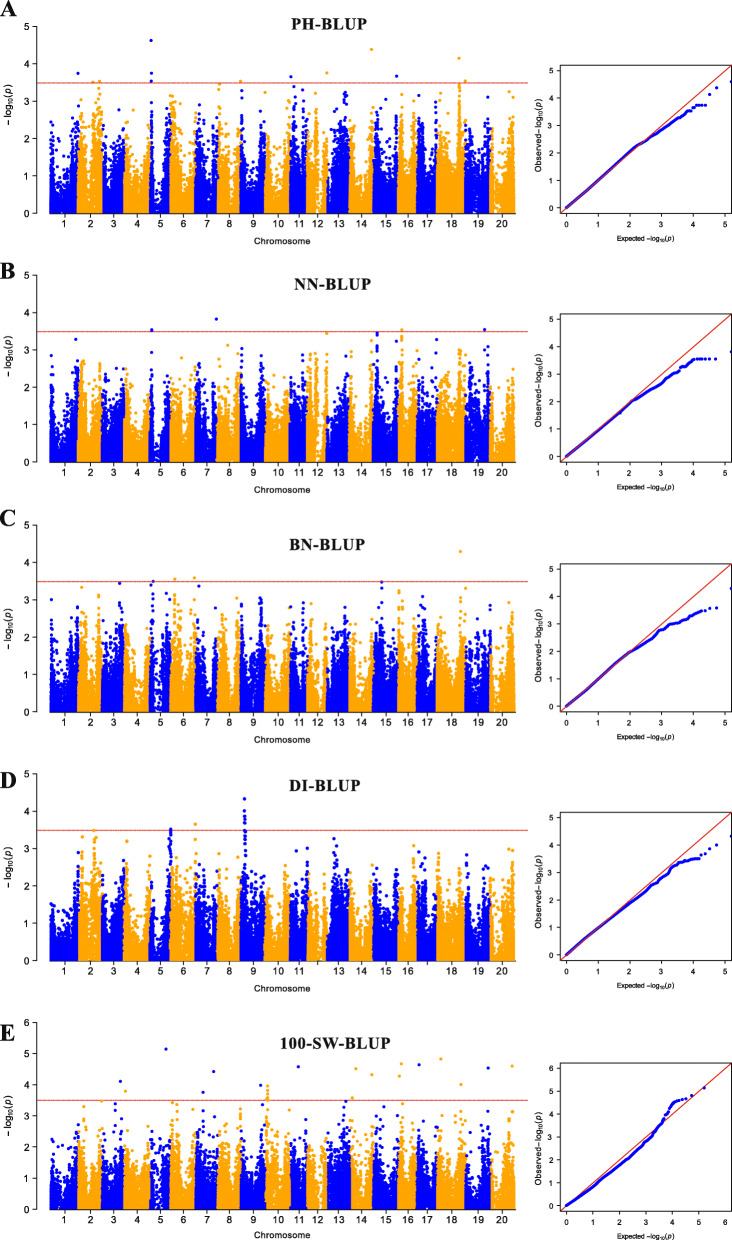
Manhattan and Q-Q plots of the GWAS for the four plant architecture-related traits and 100-SW in soybean landraces. The horizontal red line indicates the genome-wide significance threshold (−log_10_(*P*) ≥ 3.5). **a**, **b**, **c**, **d** and **e** represent association mapping of PH, NN, BN, DI and 100-SW based on the BLUP values, respectively

The MLM model, with both Q and K-matrices as covariates, was used in the association study of 82,187 SNPs with PH, NN, BN, DI and 100-SW from the 133 soybean landraces. To identify SNPs associated with the five traits, we used the MLM (Q + K) model to analyze five traits in the different environments. A total of 59 SNPs was significantly associated (−log_10_(*P*) ≥ 3.5) with five traits in at least two environments. Among them, 12, 12, 4, 4 and 27 SNPs were significantly associated with PH, NN, BN, DI and 100-SW, respectively (Fig. 3 and Table 3). For PH, 12 SNPs were detected in at least two environments. Among these SNPs, AX-90380587 and AX-90406013 were markers with larger effects and were repeatedly detected in three environments, and the contribution of a single marker to the observed phenotypic variation was 14.05–18.40% (Table 3). For NN, 12 SNPs were detected in at least two environments. Among these SNPs, AX-90387160 and AX-90317160 were markers with larger effects and were repeatedly detected in three environments, and the contribution of a single marker to the observed phenotypic variation was 13.35–19.21% (Table 3). For BN, 4 SNPs were detected in at least two environments. Among these SNPs, AX-90449770 was a larger effect marker which was repeatedly detected in three environments, and its contribution to the observed phenotypic variation was 10.71–11.51% (Table 3). For DI, 4 SNPs were detected in at least two environments. Among these SNPs, AX-90460927 was markers with larger effects and were repeatedly detected in two environments, and the contribution of a single marker to the observed phenotypic variation was 16.03% (Table 3). For 100-SW, twenty-seven SNPs were detected in at least two environments. Among these SNPs, AX-90520043 was a larger effect marker which was repeatedly detected in two environments, and its contribution to the observed phenotypic variation was 20.42–21.0% (Table 3). Based on the stability of the SNPs with significant associations in each environment and the higher phenotype variation explanations, seven SNPs (AX-90380587, AX-90406013, AX-90387160, AX-90317160, AX-90449770, AX-90460927 and AX-90520043) with large effects were selected for subsequent candidate gene prediction.

**Table 3:** SNPs significantly associated with the four plant architecture-related traits and 100-SW across multiple environments

Traits	Markers^a^	Chr.	Position	Environments^b^	-log_10_ (*P)*	*R*^2^(%)	Known QTLs^c^
PH	AX-90403529	1	56,192,858	2017DT/Mean/BLUP	3.65 ~ 4.2	16.02 ~ 17.32	
	AX-90343633	2	43,898,048	2017DT/BLUP	3.53 ~ 3.54	14.02 ~ 14.57	
	AX-90380587	5	2,724,763	2016JP/2017DT/BLUP	3.74 ~ 4.53	14.05 ~ 18.26	
	AX-90497935	5	2,820,291	2016JP/BLUP	3.53 ~ 3.76	13.3 ~ 14.98	
	AX-90498802	5	2,775,722	2017DT/Mean/BLUP	3.99 ~ 4.61	16.39 ~ 18.53	
	AX-90520578	5	2,727,221	2016JP/2017DT/BLUP	3.74 ~ 4.53	14.05 ~ 18.26	
	AX-90467414	11	1,375,175	2017DT/Mean/BLUP	3.59 ~ 3.99	13.66 ~ 16.41	
	AX-90406013	14	45,923,523	2017DT/Mean/BLUP	4.23 ~ 4.44	16.63 ~ 18.4	
	AX-90335719	15	47,490,299	2017DT/Mean/BLUP	3.52 ~ 4.08	12.5 ~ 15.48	Plant height 26–10
	AX-90456181	18	57,800,102	2017DT/BLUP	3.53 ~ 3.57	13.31–14.67	
	AX-90403639	18	44,436,699	2016JP/Mean/BLUP	3.75 ~ 4.49	12.05 ~ 15.05	Plant height 26–14
	AX-90466852	18	45,787,667	2016JP/Mean	3.53 ~ 4.56	11.19 ~ 19.21	
NN	AX-90387160	7	42,526,322	2016JP/Mean/BLUP	3.81 ~ 4.56	15.1 ~ 19.21	
	AX-90435665	11	25,260,079	2017JP/Mean	3.5 ~ 3.81	14.92 ~ 15.2	
	AX-90436094	11	25,264,410	2017JP/Mean	3.5 ~ 3.81	14.92 ~ 15.2	
	AX-90427317	12	39,270,786	2017JP/Mean	3.7 ~ 3.8	15.2 ~ 15.55	
	AX-90361359	14	45,843,392	2016JP/Mean	3.79 ~ 4.58	15.1 ~ 18.57	
	AX-90453654	14	44,683,373	2016JP/Mean	3.9 ~ 4.56	15.57 ~ 18.48	
	AX-90377223	16	6,762,918	2016JP/Mean	3.79 ~ 4.59	15.12 ~ 18.6	
	AX-90451767	16	6,715,180	2016JP/Mean	3.79 ~ 4.61	15.12 ~ 18.71	
	AX-90475022	16	6,895,355	Mean/BLUP	3.54 ~ 3.91	13.27 ~ 15.61	
	AX-90507356	16	6,751,612	2016JP/Mean	3.77 ~ 4.63	15.02 ~ 18.79	
	AX-90317160	19	38,745,810	2016JP/2017DT/Mean/BLUP	3.55 ~ 4.4	13.35 ~ 17.8	
	AX-90352912	19	45,142,445	2016JP/Mean	4.03 ~ 4.19	16.16 ~ 16.84	
BN	AX-90389449	6	7,767,192	Mean/BLUP	3.55 ~ 3.98	13.59 ~ 16.39	
	AX-90420194	6	15,358,000	2016JP/Mean	3.61 ~ 3.66	11.5 ~ 11.58	
	AX-90449770	6	48,360,017	2016JP/Mean/BLUP	3.58 ~ 3.66	10.71 ~ 11.51	
	AX-90345457	18	47,321,404	Mean/BLUP	4.29 ~ 4.45	16.47 ~ 18.18	
DI	AX-90397877	8	3,019,730	Mean/BLUP	3.77 ~ 3.8	14.1 ~ 14.7	
	AX-90460927	10	44,361,012	2016JP/Mean	4.03 ~ 4.08	16.03	
	AX-90488930	18	308,829	2017JP/Mean	3.56 ~ 4.05	13.9 ~ 16.16	
	AX-90511176	18	328,596	2017JP/Mean	3.66 ~ 4.03	14.19 ~ 16.01	
100- SW	AX-90483564	3	36,787,728	Mean/BLUP	4.11 ~ 4.42	18.13 ~ 20.04	
	AX-90435834	4	1,402,717	2017JP/BLUP	3.8 ~ 3.96	14.38 ~ 15.51	Seed weight 2–1; Seed weight 47–3
	AX-90520043	5	32,154,586	2017JP/2017DT/BLUP	4.87 ~ 5.14	20.42 ~ 21	Seed weight 36–9; Seed weight 37–12
	AX-90370125	6	5,791,933	2017JP/2017DT/BLUP	3.88 ~ 4.91	15.34 ~ 19.78	Seed weight-008; Seed weight-011
100- SW	AX-90305893	7	35,963,868	2017JP/2017DT/BLUP	4.2 ~ 4.42	16.47 ~ 16.81	
	AX-90428268	7	14,899,829	2017JP/2017DT/BLUP	3.52 ~ 3.75	10.97 ~ 11.67	
	AX-90328574	9	39,625,218	2017DT/BLUP	3.98 ~ 4.28	15.04 ~ 16.8	
	AX-90390639	10	4,423,355	2017JP/2017DT/BLUP	3.64 ~ 3.7	13.89 ~ 14.24	Seed weight 34–8
	AX-90397611	10	4,455,671	2017JP/2017DT/BLUP	3.77 ~ 3.95	14.67 ~ 15.45	
	AX-90338196	10	4,366,228	2017JP/2017DT/BLUP	3.5 ~ 3.56	10.47 ~ 11.2	
	AX-90450721	10	4,397,396	2017JP/2017DT/BLUP	3.57 ~ 3.61	10.76 ~ 11.2	
	AX-90450778	10	4,426,008	2017JP/2017DT/BLUP	3.7 ~ 3.85	14.47 ~ 15.19	
	AX-90456677	10	4,365,393	2017JP/2017DT/BLUP	3.5 ~ 3.59	10.46 ~ 11.14	
	AX-90464016	10	4,376,046	2017JP/2017DT/BLUP	3.57 ~ 3.61	10.76 ~ 11.2	
	AX-90467603	10	4,363,693	2017JP/2017DT/BLUP	3.5 ~ 3.59	10.46 ~ 11.14	
	AX-90473871	10	4,426,717	2017JP/2017DT/BLUP	3.86 ~ 3.97	15.06 ~ 15.5	
	AX-90514209	10	1,523,443	2017JP/BLUP	3.52–3.54	13.27 ~ 13.62	
	AX-90462182	11	15,778,903	2017JP/2017DT/BLUP	4.38 ~ 4.58	15.2 ~ 15.48	Seed weight 36–11; Seed weight 4–1
	AX-90463646	14	12,829,279	2017JP/2017DT/BLUP	4.28 ~ 4.51	17.43 ~ 18.23	
	AX-90481424	14	5,733,475	2017DT/BLUP	3.55 ~ 3.58	13.47 ~ 13.82	
	AX-90512978	14	45,661,649	2017JP/2017DT/BLUP	3.6 ~ 4.54	14.11 ~ 18.24	
	AX-90496773	16	1,617,227	2017DT/BLUP	4.27 ~ 4.48	16.25 ~ 17.77	
	AX-90519309	17	4,197,693	2017JP/2017DT/BLUP	3.86 ~ 4.64	15.23 ~ 19.32	
	AX-90336868	18	48,261,812	2017JP/2017DT/BLUP	3.62 ~ 4.0	14.04 ~ 15.44	
	AX-90369283	18	7,017,555	2017JP/2017DT/BLUP	4.37 ~ 4.82	17.49 ~ 18.8	Seed weight 50–4
	AX-90350838	19	45,623,416	2017JP/2017DT/BLUP	4.29 ~ 4.41	17.68 ~ 18.24	
	AX-90460297	20	44,288,532	2017JP/2017DT/BLUP	4.31 ~ 4.6	16.93 ~ 17.58	

^a^The significant SNP ID, ^b^ 2016JP, 2017JP and 2017DT represent the environments of Jiangpu in 2016, Jiangpu in 2017 and Dangtu in 2017, respectively. ^c^ Comparision of trait-marker associations identified in this study with QTLs identified in previous studies. “Mean” represents association mapping with the mean values across three environments, “BLUP” represents association mapping with the BLUP values across three environments

### Prediction of candidate genes

**Fig. 4:**
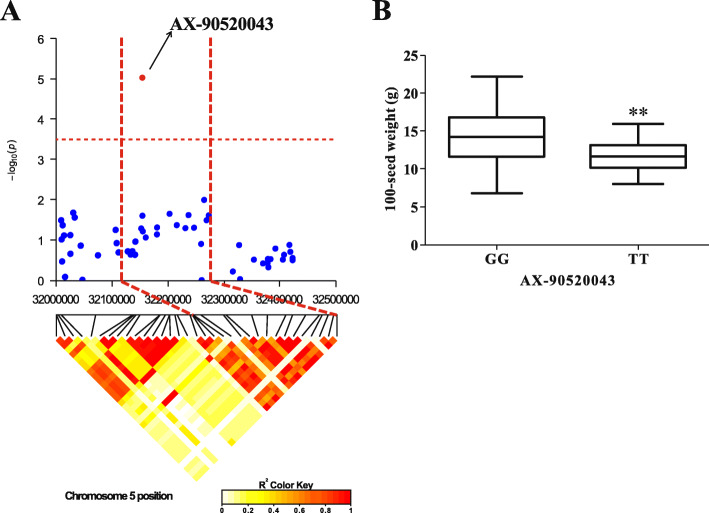
The candidate regions of the large-effect markers associated with 100-SW and phenotypic differences between accessions carrying different alleles. **a** AX-90520043 is significant associated with 100-SW, which is located on Gm05. **b** The allele effects for the 100-SW marker AX-90520043 in soybean landraces. **Significant at *P *≤ 0.01

Using haplotype analysis of the LD decay distance (± 119.07 kb) where 7 SNPs with large effects markers are located, we found that there is an LD block located in the range of 130.9 kb (32141519–32,272,444) on chromosome 5 with the SNP marker AX-90520043, which is only significantly associated with 100-SW. Compared to the candidate region where the marker AX-90520043 is located, the LD block reduces the candidate region (± 119.07 kb) by approximately 107 kb (Fig. 4a). Compared with the alternative alleles, the 100-SW of the materials carrying the favorable allele (GG) at AX-90520043 was 21.7% higher than the materials carrying the unfavorable allele (TT) (Fig. 4b). Based on the LD decay distance or the LD block and functional annotations, we selected 15 candidate genes for the four plant architecture-related traits and 100-SW in these regions near those seven SNPs with large effects. Among them, the number of candidate genes for PH, NN, BN, DI and 100-SW were four, two, one, four and four, respectively. The detailed functional annotations are shown in Table 4.

**Table 4:** Functional annotation of the potential candidate genes for the four plant architecture-related traits and 100-SW in soybean

Traits	Candidate genes	Function annotation
PH	*Glyma.05 g030900*	Pentatricopeptide repeat (PPR) superfamily protein
	*Glyma.14 g194100*	zinc finger (CCCH-type) family protein
	*Glyma.14 g194400*	Pentatricopeptide repeat (PPR-like) superfamily protein
	*Glyma.14 g194600*	ATNDI1, NDA1|alternative NAD(P)H dehydrogenase 1
NN	*Glyma.19 g128800*	PIN1, ATPIN1|Auxin efflux carrier family protein
	*Glyma.19 g129100*	TTF-type zinc finger protein with HAT dimerisation domain
BN	*Glyma.06 g294700*	GDSL-like Lipase/Acylhydrolase superfamily protein
DI	*Glyma.10 g210500*	GATA transcription factor 9
	*Glyma.10 g210600*	ARF16|auxin response factor 16
	*Glyma.10 g211000*	PIP2B, PIP2;2|plasma membrane intrinsic protein 2
	*Glyma.10 g212200*	UBC19|ubiquitin-conjugating enzyme19
100-SW	*Glyma.05 g127900*	Small nuclear ribonucleo protein family protein
	*Glyma.05 g128000*	Chlorophyll A/B binding protein 1
	*Glyma.05 g129000*	HMG-box (high mobility group) DNA-binding family protein
	*Glyma.05 g129400*	basic helix-loop-helix (bHLH) DNA-binding superfamily protein

**Fig. 5:**
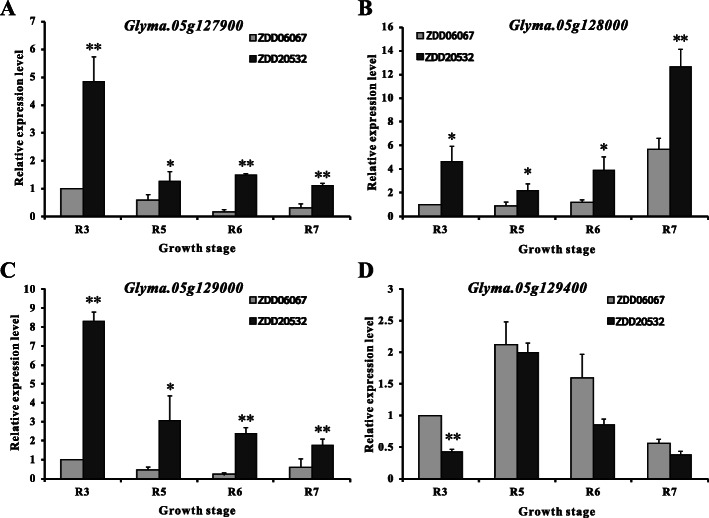
Expression analysis of potential 100-SW candidate genes in extreme materials at four growth developmental stages (R3, R5, R6 and R7). The extreme materials for 100-SW include ZDD06067 (24.36 ± 1.67 g) and ZDD20532 (4.55 ± 0.94 g). The error bar indicates the standard deviation. The results are representative of three biological replicates. *Significant at *P* ≤ 0.05; **Significant at *P* ≤ 0.01

To confirm whether the potential candidate genes participated in the accumulation of 100-SW, we tested the expression patterns of the four genes (*Glyma.05 g127900*, *Glyma.05 g128000*, *Glyma.05 g129000* and *Glyma.05 g129400*) via qRT-PCR in the seeds from the extreme materials at four developmental growth stages (R3, R5, R6 and R7). The genotype of the ZDD06067 (100-SW 24.36 ± 1.67 g) and ZDD20532 (100-SW 4.55 ± 0.94 g) extreme materials at the AX-90520043 locus were AA (unfavorable allele) and TT (favorable allele), respectively. Among the four potential candidate genes associated with 100-SW, *Glyma.05 g127900*, *Glyma.05 g128000* and *Glyma.05 g129000* showed significant differences in expression between ZDD06067 and ZDD20532 at four stages during soybean seed development (*P* ≤ 0.01) (Fig. 5). During all four tested growth stages, there was a pronounced differential expression of the 100-SW material genotype by ZDD06067 (higher) and 100-SW genotype ZDD20532 (lower). Therefore, *Glyma.05 g127900*, *Glyma.05 g128000* and *Glyma.05 g129000* may be used as candidate genes for soybean 100-SW, as they negatively regulate 100-SW in soybean.

## Discussion

The large phenotypic variations observed within the four plant architecture-related traits and 100-SW allowed us to identify the best genes with the largest effects (Table 1). In this study, the heritabilities of the five traits ranged from 65.17 to 98.66%; the smallest heritability was BN and the largest was 100-SW. The heritability of NN is approximately 40% different from that calculated by Zhang et al. (2015), but the heritabilities for other traits were not much different [5]. This may be caused by the fact that NN is greatly affected by environmental factors. In addition, the average of PH in 2017DT was higher than that in 2016JP and 2017JP, which may be due to the relatively sufficient rain in 2017DT and dry weather in 2016JP and 2017JP (Table 1). The results of previous studies confirmed that PH, NN, BN, DI and 100-SW have a crucial role in soybean plant architecture or yield [42, 43]. Correlation analysis showed that there was a significant positive correlation between PH, NN, BN and DI, while 100-SW was only significantly positively correlated with DI. This may be fact that PH, NN, BN and DI are plant architecture traits, and 100-SW is related to yield traits. Additionally, DI was significantly positively correlated with 100-SW, which indicated that the photosynthetic products of the larger stems were transported from the source to the reservoir faster, thus the flux was larger, which played an important role in the later grain and development [44]. Therefore, during the soybean breeding process, breeders should pay special attention to selecting materials with slightly higher PH and NN, moderate BN, and thicker DI to ensure high soybean yield.

In this study, the MLM (Q + K) model was used for a GWAS to examine the four plant architecture-related traits and 100-SW. Fifty-nine stable and significant SNPs were identified, of which 25 were located in QTLs of the reported related traits. Thirty-four novel loci were identified in this study. The three SNPs (AX-90335719, AX-90403639, and AX-90466852) that were significantly associated with PH were consistent with the results of Sun et al. (2006) [45]. These SNPs, which are significantly associated with NN, BN, and DI are all new loci identified in this study. Of the 27 SNPs significantly associated with 100-SW, 22 were within reported QTLs for seed weight, and 5 were new loci. The significantly associated marker AX-90435834 located on chromosome 4 is located within previously reported two seed weight QTLs [16, 46]. Both AX-90520043 and AX-90370125 are located within the previously reported seed weight related QTLs [11, 47, 48]. The 10 SNPs on chromosome 10 are close located and may belong to the same seed weight QTL which are located within previously reported seed weight QTL [49]. Both AX-90462182 on chromosome 11 and AX-90369283 on chromosome 18 are located within previously reported QTLs [48–50]. In this study, thirty-four new loci were identified and this may be related to the different populations and environments used for association mapping.

Through the functional annotation of genes, the current study predicted a total of 15 potential candidate genes associated with PH, NN, BN, DI and 100-SW. Among these 15 genes, four genes (*Glyma.05 g030900*, *Glyma.14 g194100*, *Glyma.14 g194400* and *Glyma.14 g194600*) are related to PH. The proteins encoded by *Glyma.05 g030900* and *Glyma.14 g194400* belong to the pentatrico peptide repeat (PPR) family of proteins, which are involved in the metabolic regulation of RNA, act as binding proteins, and have chloroplasts and mitochondria as their sites of action [51]. *Glyma.14 g194100* and *Glyma.14 g194600* encode a zinc finger family protein and ATNDI1, respectively. The zinc finger family protein gene is expressed in different developmental stages of different tissues of plants, regulating seed development and germination, and plays an important role in plant growth and development [52]. The potential candidate genes for NN are *Glyma.19 g128800* and *Glyma.19 g129100*, which encode an auxin transporter protein and zinc finger protein, respectively. *Glyma.06 g294700*, a candidate gene for BN, encodes a GDSL-type lipase that belongs to a large gene family in plants. Plant GDSL lipase plays an important role in plant growth and development, organ morphogenesis and lipid metabolism [53]. There are four genes related to DI, among which *Glyma.10 g210500* encodes a GAGA-binding transcription factor protein, whereas *Glyma.10 g210600*, *Glyma.10 g211000* and *Glyma.10 g212200* encode auxin response factor 16 (ARF16), a plasma membrane protein and ubiquitin-binding enzyme 19 (UBC19), respectively. Additionally, this study also predicted four candidate genes (*Glyma.05 g127900*, *Glyma.05 g128000*, *Glyma.05 g129000* and *Glyma.05 g129400*) that may be related to 100-SW. The results of qRT-PCR analysis indicated that *Glyma.05 g127900*, *Glyma.05 g128000* and *Glyma.05 g129000* were differentially expressed in 100-SW extreme materials. Among these genes, *Glyma.05 g127900* encode a ribonucleoprotein, its homologous gene is *SAD1* in *Arabidopsis*. Studies have confirmed that *SAD1* encodes a polypeptide similar to multifunctional Sm-like-snRNP proteins that are required for mRNA splicing, export, and degradation, the *sad1* mutant of *Arabidopsis* can delay seed germination [54]. *Glyma.05 g128000* encode a chlorophyll a/b binding protein, previous studies have shown that the protein can bind to photosynthetic pigments to form a light-harvesting pigment protein complex to participate in light energy transfer and play a role in plant photosynthesis [55]. In addition, AX-90520043, which is significantly associated with 100-seed weight, is located in the CDS region of *Glyma.05 g128000*. *Glyma.05 g128000* may affect the accumulation and transport of soybean dry matter by regulating photosynthetic reaction, and it is likely to participate in the regulation of seed weight in soybean. *Glyma.05 g129000* encodes a HMG-box DNA binding protein. At present, the regulatory network of HMG-box DNA binding protein is still unclear. It may be involved in the regulation of genes involved in soybean seed development, and then participate in the regulation of 100-SW in soybean. However, further evidence is needed to functionally validate this hypothesis.

In summary, our results demonstrated that the four plant architecture-related traits and 100-SW in soybean are substantially correlated with both phenotype and genotype. The utilization of the highly associated markers detected in multiple environments and the potential candidate genes could accelerate the optimization of molecular breeding and the understanding of the genetic mechanisms underlying agronomic traits.

## Conclusion

In this study, we identified 12, 12, 4, 4 and 27 SNPs associated with PH, NN, BN, DI and 100-SW, respectively, via GWAS. Most markers were located within or close to QTLs identified in previous studies. We were particularly interested in the large-effect markers AX-90380587, AX-90406013, AX-90387160, AX-90317160, AX-90449770, AX-90460927 and AX-90520043 for PH, NN, BN, DI and 100-SW, and 15 potential candidate genes that were predicted based on functional annotations. According to the expression analyses, *Glyma.05 g127900*, *Glyma.05 g128000* and *Glyma.05 g129000* are proposed as the candidate genes for 100-SW, but further investigation is needed for verification of this hypothesis. These findings shed light on the genetic basis of PH, NN, BN, DI and 100-SW, and candidate genes could be used for further positional cloning.

## Methods

### Plant materials, field trials and trait phenotyping

The germplasm for this study contained 133 soybean landraces selected from the soybean mini core collection of 23,587 soybean germplasms [56]. Those 133 soybean landraces came from 24 provinces and were distributed in four ecoregions of China as follows: the Northeast region, the North region, the Huang-huai region and the South region [41]. The experiment materials were provided by Lijuan Qiu, a researcher from the Chinese Academy of Agricultural Sciences.

One hundred and thirty three soybean landraces were planted at Jangpu (N 31.2, E 118.4) in 2016 and 2017 and at Dangtu (N 31.6, E 118.5) in 2017 with three randomized replications, with one row per plot, 40 plants per row, 10 cm between plants within each row and 50 cm between rows. 2016JP, 2017JP and 2017DT represent the environments of Jiangpu in 2016, Jiangpu in 2017 and Dangtu in 2017, respectively. The field management was performed under normal soybean production conditions. Five major plant traits, including PH, NN, BN, DI and 100-SW, were investigated. For each of the 2016JP, 2017JP and 2017DT environments, 5 plants were randomly selected for the determination of PH, NN, BN, and DI. In 2017JP and 2017DT, 100-SW determination was performed for each block. Plant height (PH, measured in cm) is the length of the cotyledonary node to the top of the plant. Number of nodes on main stem (NN) indicate the number of nodes from the cotyledonary node to the top of the main stem node. Branch number (BN) indicates the effective number of branches. The stem diameter of the main stem was measured at the third node space with a micrometer (DI, measured in mm). 100-seed weight was obtained by weighing 100 seeds immediately after drying the seeds mixed in each block (100-SW, measured in g). The names of the 133 soybean landraces used in this study and the original phenotype data were listed in Additional file 3: Table S1.

### Phenotypic data analysis

Statistical analyses of the above five traits were performed using the R software (http:/www.R-project.org) [57]. Analysis of variance (ANOVA) was performed for all traits using a general linear model. A best linear unbiased prediction (BLUP) mixed model was fit to account for the year, trial and location effects, together with their interactions. The breeding value from the mixed model was also used for association mapping as phenotypic data. Broad-sense heritability was estimated using SAS version 9.4 [58] according to the formula:

$$ {h}^2=\frac{\sigma_g^2}{\left({\sigma}_g^2+\frac{\sigma_{ge}^2}{n}+\frac{\sigma_e^2}{rn}\right)} $$

where *σ*^*2*^_*g*_ is the genetic variance, *σ*^*2*^_*ge*_ is the variance due to the G × E interaction, *σ*^*2*^_*e*_ is the residual error, *n* is the number of environments and *r* is the number of replicates within the environment. The estimates of *σ*^*2*^_*g*_, *σ*^*2*^_*ge*_ and *σ*^*2*^_*e*_ were obtained from ANOVA by considering the environment as a random effect.

### Analysis of SNP data, LD and population structure

The 133 soybean landraces had been genotyped with the 180 K AXIOM Soya SNP array previously [41]. According to MAF > 0.05 and missing data < 10%, we detected a total of 82,187 SNPs that were used for association mapping. LD was calculated using 44,838 SNPs covering the 20 chromosomes by PLINK V1.07. The pairwise LD (*r*^*2*^) among SNPs was estimated using E (*r*^*2*^) = 1/ (1 + 4*N*_*e*_*c*) [59], where *c* represents the recombination rate of Morgan units and *N*_*e*_ represents the effective population size. The LD decay rate of the population was measured as the chromosomal distance when the average *r*^*2*^ decreased to half its maximum value [23]. The STRUCTURE 2.3.4 software based on the Bayesian model was used to explore the population structure of the 133 soybean landraces based on 82,187 SNPs [41]. A total of 82,187 SNPs were employed to conduct principal component analysis (PCA) and construct a neighbor-joining phylogenetic tree using PLINK V1.07 and PHYLIP. The TASSEL V5.2.15 software was used to calculate the kinship matrix, which represents the similarity of the different pairs of SNPs between genotypes.

### Association mapping

The existence of a population structure and relative kinship in natural populations always results in a high level of spurious positives in association mapping [60]. The population structure (Q), principal component analysis (PCA) and relative kinship (K) for the panel of 133 soybean landraces have been evaluated previously [41], and their effects on associations were evaluated with the following four statistical models: (1) the GLM model (PCA); (2) the GLM model (Q); (3) the MLM model (PCA + K); and (4) the MLM model (Q + K). The quantile-quantile plots of the estimated −log_10_(*P*) were displayed using the observed *P* values from marker-trait associations and the expected *P* values from the assumption that no associations exist between the markers and traits. The model with observed *P* values closest to the expected *P* values was chosen as the optimal model to control the confounding of population structure. Using the optimal statistical model, association analyses were carried out with 82,187 SNPs for all traits using the mean and BLUP values across multiple environments and within each environment [61]. Genome-wide association study were performed by TASSEL V5.2.15. In this study, the markers above the significant association threshold of –log_10_(*P*) ≥ 3.5 was considered significantly associated with target traits.

### Prediction of candidate genes

To reduce false positives, we defined the candidate SNPs that had significant associations in at least two environments. We selected significant SNPs with large effects to search candidate genes in their candidate regions. These candidate regions were defined by the average LD decay distance or the LD blocks. The functional annotations of genes located in the candidate regions were obtained from the SoyBase database (http://www.soybase.org/). Based on the soybean genomic annotations, potential candidate genes were predicted.

In addition, for potential candidate genes predicted for 100-SW, qRT-PCR was used to analyze the expression patterns in extreme materials with large phenotypic differences. According to the phenotypic data for 100-SW in the 2017JP and 2016DT environments, the ZDD06067 (24.36 ± 1.67 g) and ZDD20532 (4.55 ± 0.94 g) materials showed stable and large phenotypic differences. Therefore, we chose them as the extreme materials and cultivated in the field. Three replicate biological samples were collected in liquid nitrogen at four stages during soybean seed development (R3 (Pod 5 mm long at one of the four uppermost nodes on the main stem with a fully developed leaf), R5 (Seed 3 mm long in a pod at one of the four uppermost nodes on the main stem with a fully developed leaf), R6 (Pod containing a green seed that fills the pod cavity at one of the four uppermost nodes on the main stem with a fully developed leaf) and R7 (One normal pod on the main stem that has reached its mature pod color)), as defined by Fehr et al. (1971) [62]. Total RNA was extracted from R3, R5, R6 and R7 seeds using an RNA Simple Total RNA kit (TIANGEN, China). cDNA was synthesized using a Prime Script™ RT Reagent Kit (TaKaRa, Japan) with a standard protocol. The qRT-PCR primers were designed with Primer Premier 5.0 and were listed in Additional file 4: Table S2. *Gmβ-tubulin* was selected as the control gene, and the qRT-PCR assays were conducted three times using a Light Cycler 480 instrument. The relative expression levels of the candidate genes were calculated using the comparative 2^−△△CT^ method [63]. Statistical analyses were performed with the Student’s *t*-test.

## Supplementary Information

**Additional file 1: Fig. S1.** Average linkage disequilibrium (LD) decay rate estimated among co-chromosome SNPs.

**Additional file 2: Fig. S2.** Population structure analysis of 133 soybean landraces.

**Additional file 3: Table S1.** Phenotypic data of the four plant architecture-related traits and 100-SW in the 133 soybean landraces.

**Additional file 4: Table S2.** Primer sequences for 100-seed weight candidate genes in soybean.

## Data Availability

The dataset and materials presented in the investigation are available from the supplementary tables and Additional file 3.

## Abbreviations

QTL: Quantitative trait locus
PH: Plant height
NN: Number of nodes on main stem
BN: Branch number
DI: Stem diameter
100-SW: 100-seed weight
GWAS: Genome-wide association study
SNPs: Single nucleotide polymorphisms
LD: Linkage disequilibrium
*h*^2^: Heritability
ANOVA: Analysis of variance
BLUP: Best linear unbiased prediction
GLM: General linkage model
MLM: Mixed linkage model
PCA: Principal component analysis
qRT-PCR: Real-time quantitative PCR
