# Supplementary figures and images for: Genome-wide association studies of plant architecture-related traits and 100-seed weight in soybean landraces
Source: BMC Genom Data. 2021 Mar 6;22:10. doi: 10.1186/s12863-021-00964-5 (PMC7937308; doi:10.1186/s12863-021-00964-5)

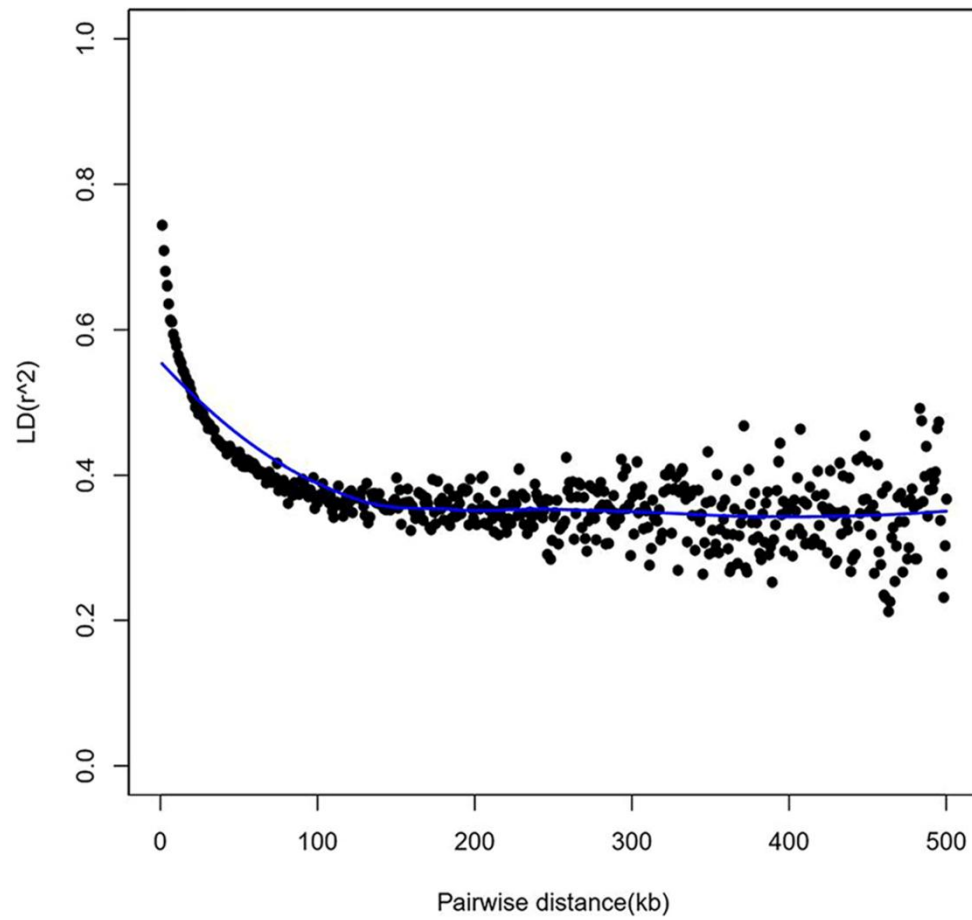

**Figure S1 Average linkage disequilibrium (LD) decay rate estimated among co-chromosome SNPs.**

Supplement: Supplementary file 1 — Additional file 1: Fig. S1. Average linkage disequilibrium (LD) decay rate estimated among co-chromosome SNPs. [file 12863_2021_964_MOESM1_ESM.pdf]
