# Supplementary material for: Genome-wide association studies of plant architecture-related traits and 100-seed weight in soybean landraces
Source: BMC Genom Data. 2021 Mar 6;22:10. doi: 10.1186/s12863-021-00964-5 (PMC7937308; doi:10.1186/s12863-021-00964-5)
Supplement: Supplementary file 2 — Additional file 2: Fig. S2. Population structure analysis of 133 soybean landraces. [file 12863_2021_964_MOESM2_ESM.pdf]

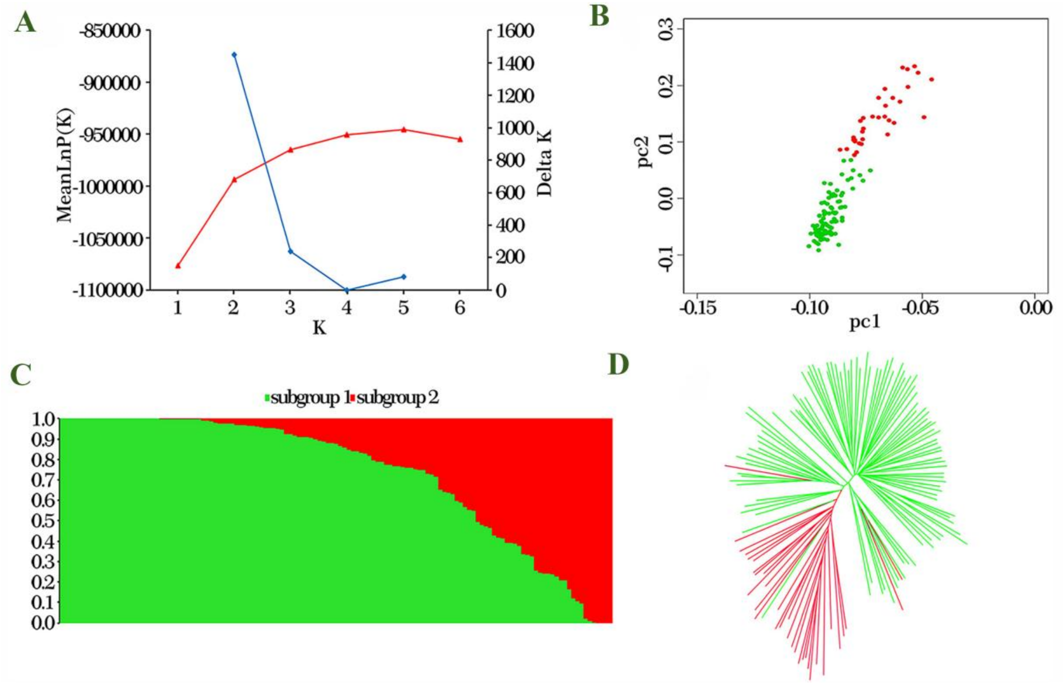


**Figure S2 Population structure analysis of 133 soybean landraces. (A) The mean Ln*P*(*K*) and Delta *K* values when *K* ranges from 1 to 6. (B) Two-dimensional scatter plot of PCA, the green dot represents subgroup 1 and the red dot represents subgroup 2. (C) Population structure of 133 soybean landraces, there are two colored segments and eachsegment represents the percentage of the individual in the population. (D) A neighbor-joiningtree of the 133 soybean landraces that can be divided into two subgroups.**
